# Debridement without bone grafting prevents osteolytic lesions progression in revision THAs with prosthesis revised

**DOI:** 10.3389/fsurg.2022.925940

**Published:** 2023-01-06

**Authors:** Keyu Kong, Fupeng Li, Hua Qiao, Yongyun Chang, Yi Hu, Huiwu Li, Jingwei Zhang

**Affiliations:** Shanghai Key Laboratory of Orthopaedic Implants, Department of Orthopaedic Surgery, Shanghai Ninth People's Hospital, Shanghai Jiaotong University School of Medicine, Shanghai, China

**Keywords:** bone grafting, osteolytic lesions, revision total hip arthroplasty (rTHA), osteolysis progression, debridement

## Abstract

**Background:**

Bone defects in revision total hip arthroplasties (rTHAs) caused by osteolysis are routinely treated with autografts or allografts, despite their various disadvantages. Currently, little is known about the prognosis of ungrafted cavities with complete debridement following prosthetic revision in rTHAs with component loosening, as few reports have focused on the application of debridement without bone grafting in osteolytic lesions that do not compromise structural stability in revision THAs with revised components.

**Methods:**

In this study, 48 patients receiving rTHAs with components revised for aseptic loosening with osteolysis between 2015 and 2019 were included. Anteroposterior and lateral radiographs of hips before and after revision surgery and last follow-up were compared to measure whether the size of the debrided osteolytic cavity without bone graft had changed.

**Results:**

In total, 48 patients with 59 osteolytic lesions were enrolled. The mean follow-up period was 3.33 years (range 2–6 years). None of the 59 cavities had progressed at the last follow-up, and 11 (18.6%) regressed. Two patients underwent re-revision according to dislocation during follow-up.

**Conclusion:**

In rTHAs with revised components, osteolytic lesions that do not influence structural stability could be debrided without grafting to avoid the disadvantages of grafting. Debridement and component revision are sufficient to prevent the progression of osteolytic lesions during surgery, without having adverse effects on the short-to mid-term prognosis.

## Introduction

The number of revision total hip arthroplasties (rTHAs) is increasing year by year (1, 2). The reasons for rTHAs include aseptic loosening, infection, and dislocation, among which aseptic loosening caused by osteolysis is one of the most common (3, 4). RTHA is a challenging procedure; if performed improperly, patients may need to undergo a second revision (5). Correcting the osteolytic cavity during surgery is of great significance to the success of the operation and long-term prognosis (6). Owing to factors such as chronic wear, uneven alignment, and outdated design, the interface friction between the femoral head prosthesis and acetabulum prosthesis can produce worn polyethylene or metal particles (7, 8). These particles are scattered throughout the effective joint cavity by the flow of the joint fluid, resulting in the release of cytokines and triggering a series of inflammatory immune cascade reactions following their phagocytosis by macrophages (9–11). The activation of osteoclasts leads to bone absorption and granuloma formation (12). Research has shown that osteolysis is still one of the main reasons patients undergo revision (4, 13). At present, related research has mostly focused on the effectiveness and long-term survival of grafting in the treatment of osteolytic lesions with well-fixed prosthesis retention, which has good clinical outcomes (14). However, some studies (15) have shown unsatisfactory incorporation between the grafts and the host bone.

Not all lesions affect prosthesis stability. Small cavities that exert insignificant influence on the osteointegration interface between the host bone and prosthesis will not affect prosthesis stability following correct intraoperative trial fit verification (16). During complete rTHA, lesions that affect prosthesis stability are reconstructed with bone grafts, augments, or jumbo cups. However, few studies have focused on the treatment of bone defects that do not affect the stability of the prosthesis and do not require reconstruction. Grafting is routinely performed in clinical practice. However, bone grafting has some inherent disadvantages. Some studies have pointed out that the implanted graft may not integrate with the original bone tissue, and may even become a new source of wear particles, thus further accelerating the occurrence and progression of osteolysis (17). In addition, allogeneic bone also faces problems such as high costs and shortage in supply (18, 19). Currently, there are few stable allogeneic bone sources that patients can afford in clinical practice (19). More importantly, bone grafts also carry the risk of infection and immune rejection when adopting allogeneic bone, which delays bone healing and increases the risk of re-revision (17, 20). Similarly, autogenous bone grafts also face problems such as limited bone quantity, numbness, and pain after bone extraction (17).

Consequently, the prognosis of mere debridement without grafting in small osteolytic lesions is significant as this alternative treatment can avoid the disadvantages and economic burden of bone grafting if osteolysis is blocked. However, to the best of our knowledge, no studies have yet reported such a prognosis; thus, we conducted retrospective study to explore this topic.

This study aimed to discover (1) how debridement alone will affect the size of osteolysis lesions that do not need reconstruction in revision THAs with revised components; (2) whether simple debridement could block the cascade reaction of osteolysis; (3) whether debridement without bone graft affects the integration of prosthesis and bone; and (4) whether this method would affect the stability of prosthesis due to the progression of osteolysis.

This study was reported in accordance with the TREND reporting checklist.

## Methods

### Study participants

This study was conducted in accordance with the Declaration of Helsinki (revised in 2012), and was approved by the Ethics Committee of the Shanghai Ninth People's Hospital, Shanghai Jiao Tong University School of Medicine. Informed consent was obtained from all participants. All 310 subjects who underwent revision hip arthroplasty between January 2015 and June 2019 were retrospectively reviewed. The inclusion criteria were as follows: (1) The patients were diagnosed with periprosthetic osteolysis according to the standards of DeLee and Charnley (21). (2) Patients underwent revision THAs for aseptic loosening. (3) Debridement without bone grafts was applied to small lesions, and all related components were revised during surgery. The exclusion criteria were as follows: (1) Patients who did not agree to participate in the study. (2) Patients who received revision THAs for infection, periprosthetic fractures, or other reasons. (3) Patients who were lost to follow-up or who had a follow-up of less than 2 years.

**Figure 1:**
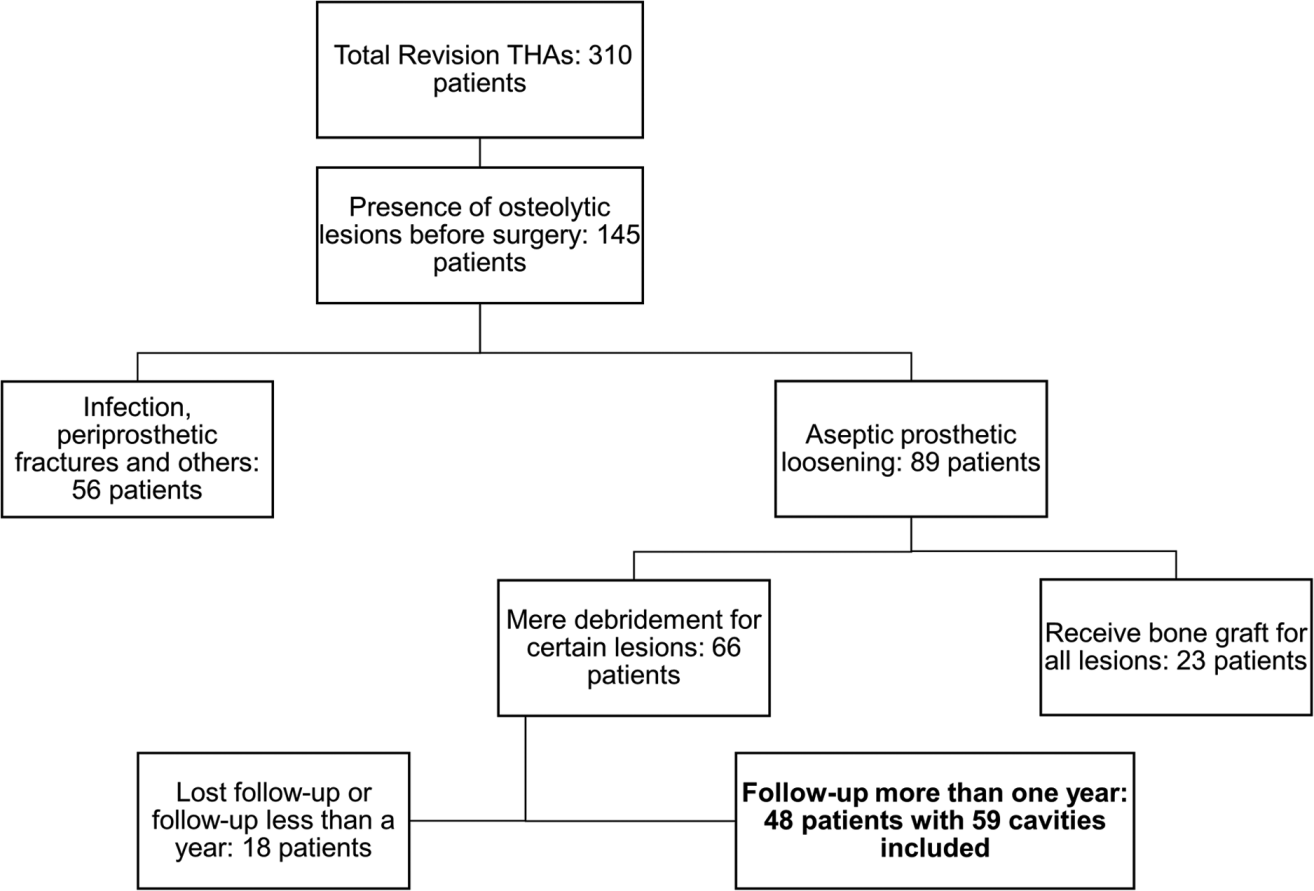
A flowchart to illustrate our inclusion criteria and results.

Of the 310 revision cases, 145 were classified as having osteolytic lesions before revision based on preoperative radiographs. Among them, 89 patients were diagnosed with aseptic loosening, while 56 underwent surgery for infection, periprosthetic fractures, and other reasons. Among the 89 candidates, 23 patients received bone grafts of all detectable osteolytic lesions during revision surgery, and 18 were lost to follow-up or had a follow-up period of less than 1 year. Finally, 48 patients with 59 osteolytic cavities treated using debridement alone with a follow-up period of at least 2 years were included in our study. The strategy of study participant selection is shown in Figure 1.

### Radiographic measurement and evaluation

**Figure 2:**
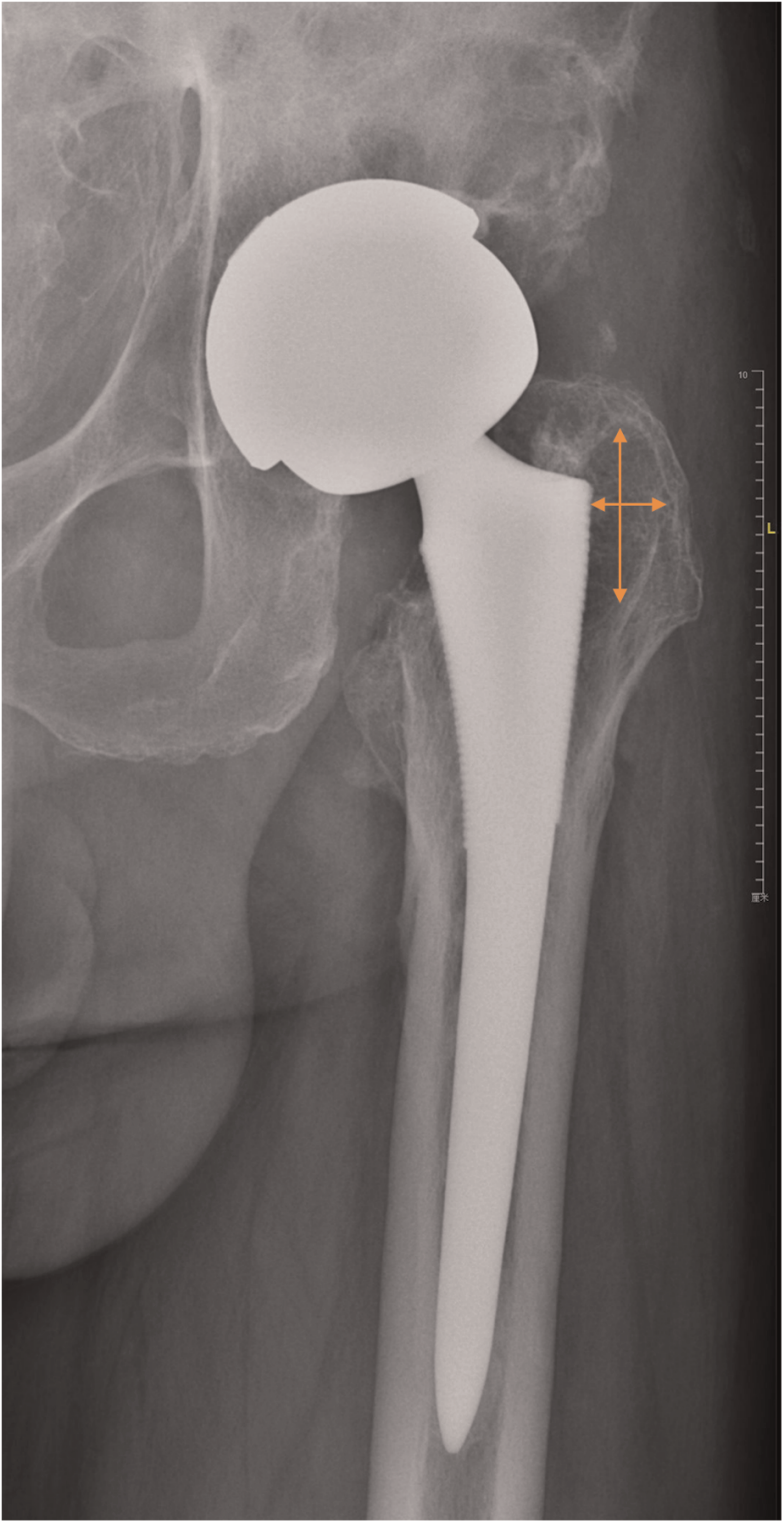
An example to illustrate our measuring approach. Two lines with arrows represent two longest diameters in the horizontal and vertical axes, respectively. Lesion size (cm^2^) equals the product of two diameters.

**Figure 3:**
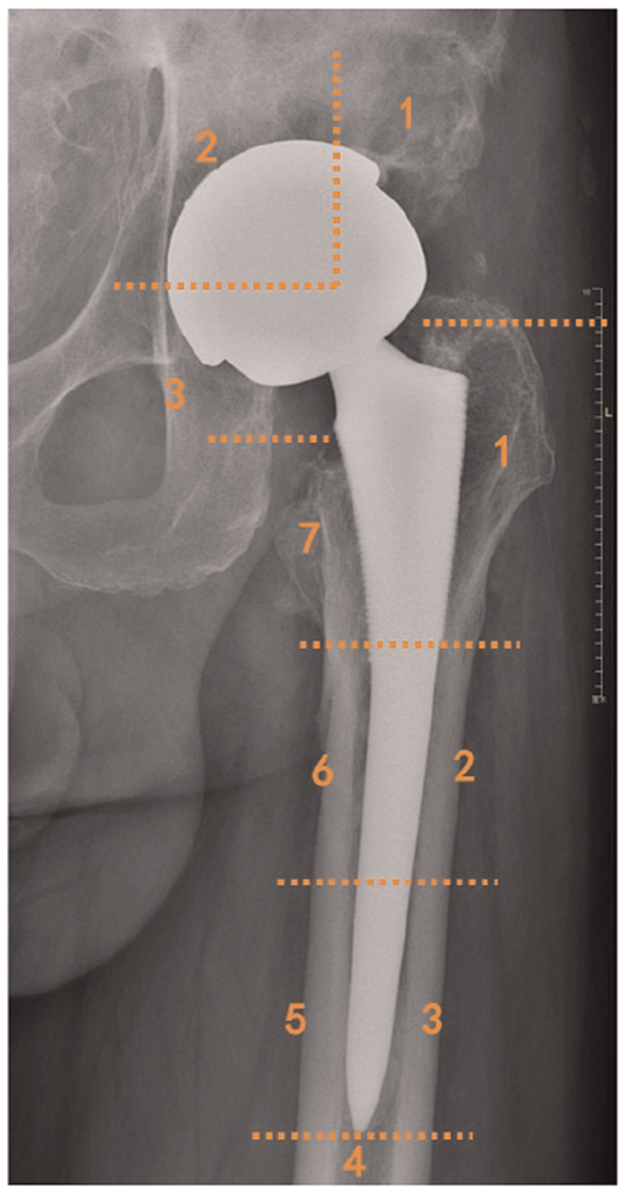
Illustration of femoral and acetabular zone classification by Gruen and by DeLee and Charnley.

After the patient's visit to the hospital, radiographs of the anteroposterior and lateral views were first obtained for the affected hip. In addition, anteroposterior and lateral radiographs were obtained immediately after surgery and at each follow-up at 3 weeks, 6 weeks, 3 months, 6 months, 1 year, and annually thereafter. Three doctors identified and evaluated the borders of the osteolytic lesions in a blinded manner. The size of the osteolytic lesions was measured in the anteroposterior x-ray as the longest diameter of the lesion in the horizontal axis multiplied by that in the vertical axe (22) (Figure 2). To determine whether the osteolytic process was blocked, we compared the latest follow-up lesion size to the initial postoperative lesion size. According to the evaluation standard of Min et al. (23), if the lesion size increased by more than 50%, or was greater than 1 cm^2^, the osteolytic process was defined as having progressed. If lesion size decreased by more than 50% or was greater than 1 cm^2^, it was defined as regressed. Osteolysis with a size change of <50% and less than 1 cm^2^ was defined as stabilized (23). The locations of the femoral and acetabular osteolytic lesions were identified according to the Gruen zone classification (24) and DeLee and Charnley zone classification (21), respectively (Figure 3). All the lesions around the femoral component were located at zone 1 and zone 7. Cup loosening was defined as described in a previous study (25). The migration of prostheses was determined by comparing radiographs taken at the last follow-up with those taken immediately postoperatively. Definite loosening was defined as an acetabular migration of ≥2 mm, with implant rotation or screw breakage. Probable loosening was defined as a radiolucent line >1 mm in all three acetabular zones without any signs of migration, rotation, or screw breakage. Osteointegration was evaluated using the criteria described by Moore et al. (26).

### Statistical analysis

Statistical analysis was conducted using SPSS version 19.0 for Windows (Inc., Chicago, IL, United States). Quantitative data with normal distribution are presented as means with ranges, and categorical variables are presented as percentages.

## Results

### Demographic information of patients and their osteolytic lesions

We enrolled 48 revision patients with 59 osteolytic lesions in this study. The patients had a mean follow-up time of 3.33 years, and 30 (62.5%) received revision surgery on the right side. Among the 59 ungrafted osteolytic cavities, 39 (66.1%) were located in zone 1 of the femur according to the Gruen zone classification. Other detailed demographic information of the patients and lesions, such as age at surgery and sex, are shown in Tables 1, 2.

**Table 1:** Patient demographic background information.

Demographic	Overall (*n* = 48)
Gender, *n* (%)	
Female	25 (52.1%)
Male	23 (47.9%)
Age at surgery, mean (range)	63.92 (30–86)
Follow-up period, mean (range)	3.33 (2–6)
Revision hip side, *n* (%)	
Left	18 (37.5%)
Right	30 (62.5%)

**Table 2:** Locations of osteolytic cavities according to Gruen classification (femur) and DeLee and Charnley classification (acetabulum).

Osteolytic cavity location	Overall (*n* = 59)
Femur, Gruen, *n* (%)	47 (79.7%)
Zone 1	39 (66.1%)
Zone 7	8 (13.6%)
Acetabulum, DeLee and Charnley, *n* (%)	12 (20.3%)
Zone 1	3 (5.1%)
Zone 3	9 (14.2%)

### Change in lesion size during the follow-up period

**Figure 4:**
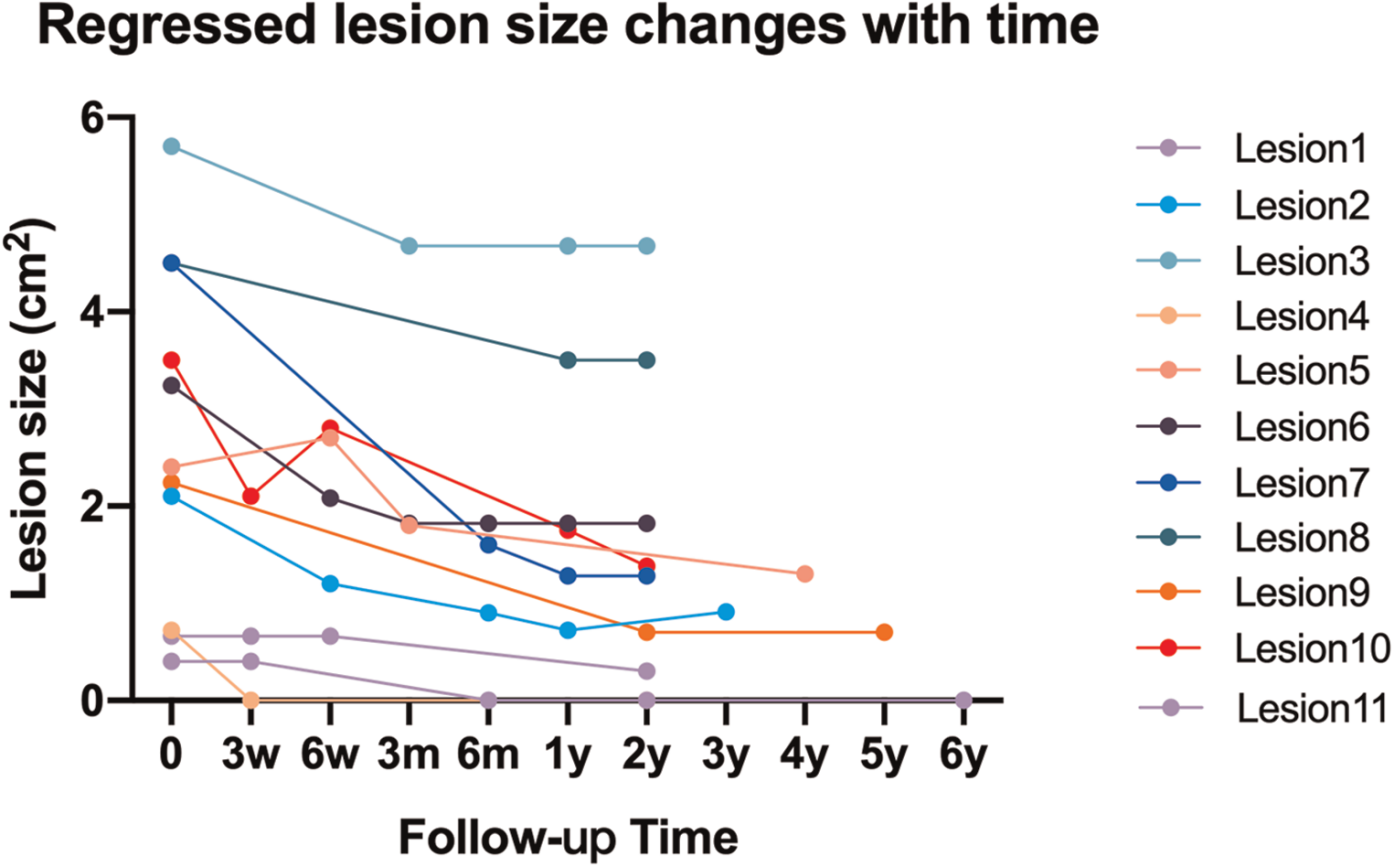
Line chart to show time-dependent change of lesion size in 11 regressed osteolytic lesions.

Based on the previously listed criteria, during the 2-year-minimum follow-up, 11 cavities (18.6%) regressed, while the other cavities remained stable. The mean lesion size of the 39 cavities in zone 1 of the femur reduced from 2.82 cm^2^ immediately after operation to 2.31 cm^2^ at the last follow-up (Table 3). The time-dependent change in lesion size of the 11 regressed cavities is illustrated in Figure 4.

**Table 3:** Comparison of lesion sizes between immediate postoperation and last follow-up and the proportion of regressed lesions and stable lesions, respectively.

Lesion size (cm^2^)	Postop, mean (range)	Last follow-up, mean (range)	Regressed, *n* (%)	Stable, *n* (%)
Femur, zone 1	2.82 (0.18–15.12)	2.31 (0–14.4)	8/39 (20.5%)	31/39 (79.5%)
Femur, zone 7	1.19 (0.15–2.24)	0.93 (0–2.09)	1/8 (12.5%)	7/8 (87.5%)
Acetabulum, zone 1	0.80 (0.36–1.20)	0.58 (0.20–0.99)	0/4 (0.0%)	4/4 (100.0%)
Acetabulum, zone 3	2.16 (0.43–5.70)	1.55 (0.31–4.68)	2/8 (25.0%)	6/8 (75.0%)

### Short- to mid-term prognosis of ungrafted osteolytic lesions in revision THAs

In addition to the lesion size change, we also focused on the short-term prosthesis stability and integration between the bone and prosthesis. At the last follow-up, all the components remained stable without prosthesis migration. None had new circumferential radiolucent lines or lesions at the last follow-up. Two patients underwent re-revision for dislocation, and the overall survival of our revision THAs was 95.8% (46/48).

## Discussion

The incidence of osteolytic cavities is high in revision patients (3), and it is generally accepted that bone graft or metal reinforcement implantation is required to reconstruct the acetabulum for large-scale osteolysis and bone defects that affect structural stability (27–29). Furthermore, whether debridement without bone grafting can be applied in osteolytic cavities that do not affect prosthesis installation and initial stability after related prosthesis revision remains controversial.

Bone grafting is a common surgical method. In a prior study, Verspeek et al. (30) followed 86 hip revision patients for 15 years and found that bone grafts could significantly reduce the risk of re-revision and osteolysis. However, they have inherent disadvantages. Gamradt and Lieberman (17) stated in their research that extravasation during operation may be a source of third-party wear particles, which could have negative effects on bone grafts. In addition, there are still several limitations, such as limited sources and the high cost of bone grafts. According to Beswick and Blom (31), the cost of 1 g of allogeneic cancellous bone is 78–86 US dollars, and even the cost of calcium phosphate cement is 26 US dollars per gram. Furthermore, applied bone grafts are prone to infection. Lee et al. (32) previously reported that in the follow-up of 140 patients with bone grafts, 7.8% developed bone graft infection. Allogeneic bone also has the possibility of self-rejection, and the osteoinduction ability of allogeneic bone itself is damaged by irradiation.

On the other hand, there are also disadvantages to not receiving a bone graft. In theory, osteolysis is a cascading process. Macrophages release inflammatory factors and activate the downstream NF-κB signaling pathway following phagocytosis of foreign particles (such as those released by grafts), and other extensively verified pathways are activated to amplify this signal (33). Whether osteolysis continues to occur if this pathway cannot be completely blocked remains unclear. Owing to the complex shape of bone defects, in some revision surgeries, some osteolytic areas may not be completely cleaned. This is particularly true when the components are retained. In contrast, more complete debridement is performed when components are revised during revision THAs. Few studies have reported the prognosis of ungrafted lesions with prostheses that do not affect structural stability, which is one reason why this study was conducted.

In revision THAs, the bone defect with a small scope, which does not affect prosthesis installation or initial stability during operation, was treated by complete debridement following component removal without bone graft in our hospital. The follow-up results showed that the dissolution area would not continue to expand if complete debridement was performed. Our results provide convincing evidence that complete debridement is an effective alternative to handle small lesions. In addition, our study included patients whose acetabular or femoral components were revised during revision to expand the scope of our conclusion, as complete debridement minimizes the number of particles and further decreases the occurrence of recurrent osteolysis.

This further shows that even if in cases with osteolytic tissue that is invisible to the naked eye or cannot be thoroughly cleaned due to other reasons, when the prosthesis with a large number of wear particles is replaced, osteolysis is blocked after the majority of osteolytic tissue is cleaned. Mochida et al. (34) showed that the concentration of wear particles around the prosthesis is proportional to the degree of osteolysis. In addition, Kobayashi (35) quantitatively analyzed the diameter and number of wear particles around the prosthesis in 18 patients following joint replacement and found that osteolysis was more likely to occur when the number of wear particles per gram of tissue was greater than 10^10^. Based on the above research, we believe that the residual wear particles were insufficient to initiate the osteolysis process after debridement of the main osteolytic tissue during the operation. In our study, no osteolysis progressed, which indicates that complete debridement following prosthesis removal blocked the recurrence of osteolysis because it minimized the number of particles.

Our research reports the short-to mid-term follow-up of non-grafted patients, showing a similar failure rate compared with that reported in grafted patients in a prior study by Villatte et al., indicating that non-grafting exerts no adverse effect on the short-to mid-term prognosis of patients (36). Moreover, the two re-revision patients in our study underwent surgery for dislocation of the prosthesis, which has little to do with the remaining cavities in the femur or acetabulum.

Nevertheless, this study has several limitations that should be considered. First, we did not compare the lesion size between the patients without bone graft and those with bone graft after component removal. This setting is due to the fact that in recent years, our hospital has rarely performed bone graft treatment for small cavities. It is obviously inappropriate to compare the prognosis of a huge grafted cavity with the small cavity discussed in this paper, and the follow-up time is relatively short. If osteolysis progression is not blocked after revision and continues to worsen, 2 years is sufficient to observe significant changes. Furthermore, if the osteolysis cavity is enlarged during long-term follow-up, it is difficult to judge whether this may be due to the continuous progression of residual osteolysis during the last operation or the new osteolytic lesions caused by the generation of new wear particles. We will continue to follow-up these patients and update our study at an appropriate time point. Finally, the number of patients included in the study was limited. We will continue to pay attention to, and include, such in future, larger patients, and will update the research results in a timely manner.

## Conclusion

For some osteolytic cavities that do not affect structural integrity after prosthesis revision, debridement can be performed without bone grafting. This treatment can not only avoid the defects of allogeneic bone and autogenous bone grafts but also block the occurrence and progression of osteolysis.

## Data Availability

The raw data supporting the conclusions of this article will be made available by the authors, without undue reservation.
